# Association between Kaposi’s sarcoma-associated herpesvirus genotype and clinical types

**DOI:** 10.3389/pore.2025.1612009

**Published:** 2025-04-28

**Authors:** Shohei Yogi, Haruna Ishikawa, Aya Oshiro, Reo Yamazato, Chiharu Sakamoto, Yasuka Tanabe, Karina Uehara, Kiyoto Kurima, Shinichiro Kina, Kenzo Takahashi, Hirofumi Arakawa, Takao Kinjo

**Affiliations:** ^1^ Division of Morphological Pathology, Department of Basic Laboratory Sciences, School of Health Sciences, University of the Ryukyus, Okinawa, Japan; ^2^ Department of Pathology, University of the Ryukyus Hospital, Okinawa, Japan; ^3^ Department of Medical Education and Development, Graduate School of Medicine, Gunma University, Maebashi, Gunma, Japan; ^4^ Department of Dermatology, Graduate School of Medicine, University of the Ryukyus, Okinawa, Japan; ^5^ Division of Cancer Biology, National Cancer Center Research Institute, Tokyo, Japan

**Keywords:** KSHV, genotype, K1, immune response, Kaposi’s sarcoma

## Abstract

Kaposi’s sarcoma (KS) is a vascular intermediate malignant tumor classified into four clinical types: classic, AIDS-related, iatrogenic, and endemic. Kaposi’s sarcoma-associated herpesvirus (KSHV) is the causative agent of KS. Six KSHV genotypes (A, B, C, D, E, and F) classified by K1 or two genotypes (P and M) by K15 have been reported. However, whether the KSHV genotype affects clinical presentation remains elusive. Herein, we investigated the association between viral genotypes and clinical presentations in patients with KS in Okinawa, an endemic area in Japan. Classic KS caused by KSHV genotype C was identified as the most common clinical type of KS in Okinawa. Conversely, 80% of the patients with AIDS-related KS were associated with genotype A. According to K15 genotyping, the population of genotype M was higher than that of genotype P. Although genotype M accounted for most cases of both classic and iatrogenic KS in Okinawa, genotype P constituted the majority of AIDS-related KS. Regarding the association between the K1 and K15 genotypes, single genotype A was associated with genotype P, whereas single genotype C was associated with genotype M. These K1 and K15 associations in Okinawa differed from those in Europe and Africa. In terms of the association between viral genotype and clinical types, A/P tended to be associated with AIDS-related KS and genotype C/M tended to be associated with classic KS. The findings of the current study suggest that the KSHV genotype in Okinawa differs from that in other countries, which is related to the KSHV geographic distribution and population migration. Our data also suggest that the viral genotype in Okinawa is associated with clinical presentations.

## Introduction

Kaposi’s sarcoma (KS) is an intermediate malignant soft tissue tumor that develops in the skin, lymph nodes, and visceral organs. Four clinical types of KS have been identified: AIDS-related, classic, endemic, and iatrogenic [1–3]. AIDS-related KS occurs in patients with AIDS as an opportunistic infection characterized by its aggressive presentation. Patients develop multiple cutaneous and visceral lesions that spread rapidly. Classic KS typically affects older individuals manifesting as multiple cutaneous lesions in the extremities; however, the lesions are usually limited to the skin and rarely involve the visceral organs. Skin lesions in classic KS gradually grow and occasionally undergo spontaneous regression [1, 2].

In 1994, Chang et al. isolated Kaposi’s sarcoma-associated herpesvirus (KSHV) from KS lesions [4]. The viral genome is 160–170 kb in size [5], with more than 90 open reading frames (ORFs) [6]. Given that the KSHV ORF K1, located at the left end of its genome, has highly variable sequence polymorphism, it has been used for KSHV genotyping. Molecular analysis of K1 has defined six KSHV genotypes: A, B, C, D, E, and F [7–10]. K1 encodes a transmembrane protein with 289 amino acids, that constitutively activates the downstream Akt and NF-κB pathways [3, 6, 11–13]. Through these signal transduction pathways, K1 has been shown to possess transformation capability, both *in vivo* and *in vitro*, and is thus regarded as an important viral oncogene [11–13]. In addition to ORF K1, ORF K15, located at the right end of the genome, exhibits sequence variability and has been used to determine genotypes, such as prototype (P) and minority (M). Although the geographical prevalence of K15 genotype has been reported, data from Asia remain limited [14–16].

Different KSHV genotypes have been reported to exhibit distinct clinical presentations [17–19]. For example, Mancuso et al. revealed that patients with genotype A show rapid development of KS, whereas those with genotype C exhibit slow progression [17]. Zhang et al. found that genotype A caused mucosal lesions more frequently than genotype C [19]. According to a study exploring the genetic diversity of KSHV genotypes in South Africa, genotype A (subtype A5) was linked to more extensive KS lesions in patients with AIDS [18]. In our previous study conducted in Okinawa, the southwest islands of Japan, which is recognized as an endemic area for classic KS, we analyzed the K1 gene sequence in classic and AIDS-related KS, which were proved to be genotypes C and A, respectively [20]. *In vitro* studies revealed that the transformation activity of genotype A is more potent than that of genotype C [13]. These findings suggest that viral genotypic differences may be related to clinical presentation. However, the relationship between the KSHV genotype and clinical features remains unknown, and a case series study of KS targeting the association between the KSHV genotype and clinical presentation has not been conducted. In the current study, we examined the KSHV genotypes in Okinawa, an endemic area in Japan, and evaluated the association between KSHV genotypes and clinical features.

## Patients and methods

### Patients with KS in Okinawa

A total of 41 patients with KS diagnosed between 2006 and 2023 were identified from the surgical pathology records of the University of the Ryukyus Hospital, Okinawa, Japan. A pathological diagnosis system and electronic medical records were used to retrieve clinical information and pathological findings. Based on WHO classification of skin lesions in KS, the clinical stage was classified as either patchy/plaque or nodular [21]. The basic characteristics of patients with KS are summarized in Table 1, and laboratory data are shown in Supplementary Table S1. This study was approved by the institutional review boards of the University of the Ryukyus, Okinawa, Japan (No. 15-843-03-03-01). Patient consent to participation is not applicable.

**TABLE 1:** Characteristics of patients with Kaposi’s sarcoma in Okinawa.

Clinical type		Classic	AIDS-related	Iatrogenic	Unknown
Number of cases (%)		18 (43.9)	10 (24.4)	12 (29.3)	1 (2.4)
Age	Average [95% CI]	80.4 [75.4–85.4]	39.2 [32.4–46.0]	74.7 [70.0–79.4]	58.0
Sex	Male	14	10	9	1
	Female	4	0	3	0
Lesions	Skin	18	7	11	1 (penis)
	Other (area)	0	3 (lung, lymph node, colon)	1 (lymph node)	0
Origin	Miyako	9	0	6	0
	Okinawa island	9	9	6	0
	The other	0	1	0	0
K1 genotype	A	0	5	1	0
	C	13	2	5	1
	A + C	4	3	4	0
	D	1	0	2	0
K15 genotype	P	2	7	1	1
	M	9	2	5	0

### PCR amplification from formalin-fixed, paraffin-embedded (FFPE) samples

To determine the viral genotype, genomic DNA extracted from FFPE samples was subjected to KSHV PCR using a ReliaPrep FFPE gDNA Miniprep System (Promega, Madison, WI, USA). The variable regions of K1, including VR1 and VR2, were amplified by PCR using the PrimeSTAR Max DNA polymerase (Takara Bio, Shiga, Japan). The primers K1 VR1 F (5′-TGC​CAA​TAT​CCT​GGT​ATT​G-3′), K1 VR1 R (5′-CAC​AAG​GTT​TGT​AAG​ACA​GG-3′), K1 VR2 F (5′-CGC​GTT​GTG​CCA​ATA​TAA​CT-3′) and K1 VR2 R (5′-TGG​TTC​CTA​TCA​GAG​CTA​CG-3′) were used to amplify VR1 and VR2. K15 was also analyzed to define P (prototype) or M (minority) genotypes using nested PCR [15]. To detect the K15 (P) genotype, K15P-OF (5′-TGC​AGG​CTT​GGT​CAT​GGG​TTA​C-3′) and K15P-OR (5′-GGGACCACGCYGCAATTAAATG-3′) were used for first PCR, and K15-3C (5′-ACG​CAT​ACA​TGT​ACT​GCC​AC-3′) and K15-4C (5′-CTT​TGA​TAT​TGC​CAG​TGG​TG-3′) were used to perform nested PCR. To detect the K15 (M) genotype, K15M-OF (5′-TGT​TGG​TTG​CAA​TGC​TTA​GGT​G-3′) and K15M-OR (5′-GCC​TTT​GCC​AGT​TGG​AGT​TTC-3′) were used for the first PCR, and LGH 2473 (5′-CAT​GCA​GCG​AGC​TTG​AGA-3′) and LGH 2474 (5′-CTT​TGA​GTA​CTG​TTT​GTG-3′) were employed to perform nested PCR.

### DNA sequencing

PCR products were purified using the Fast Gene Gel/PCR Extraction Kit (Nippon Genetics, Tokyo, Japan). After cycle sequencing reaction using the BigDye Terminator v3.1 Cycle Sequencing Kit (Thermo Fisher Scientific, Waltham, MA, USA), the DNA sequence was determined using a 3500 Genetic Analyzer (Thermo Fisher Scientific).

### Statistical analysis

Data were analyzed using a one-way ANOVA, followed by Bonferroni’s multiple comparison test. Fisher’s exact test was used to evaluate differences between groups. Statistical significance was set at p < 0.05.

## Results

### General characteristics of patients with KS in Okinawa during the study period

**FIGURE 1:**
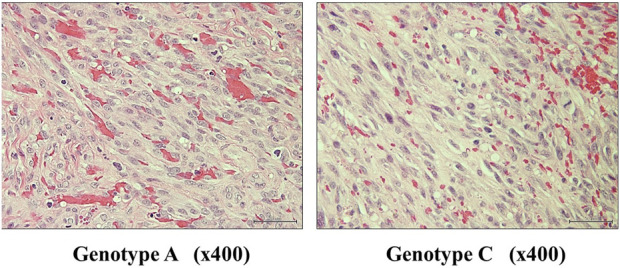
Representative histological images of KS lesions from genotypes A and C. Lesions of KS show several small slit-like vessels and proliferation of spindle tumor cells around the vessels. Histological findings appear similar among the clinical types and KSHV genotypes. KS, Kaposi’s sarcoma; KSHV, Kaposi’s sarcoma-associated herpesvirus.

Table 1 presents the general characteristics of patients with KS in Okinawa, with a total of 41 cases identified from 2006 to 2023. Among them, 18 patients had classic KS (44%), indicating that classic KS was the most prevalent clinical type, followed by iatrogenic KS (29%). Half of the patients with classic or iatrogenic KS were from the Miyako islands, located in Okinawa prefecture, where classic KS is known to be endemic [22]. Ten patients with AIDS-related KS were identified (24%); all were homosexual males with an average age younger than the other clinical types (p < 0.01). However, all clinical types of KS are more common in males than females. Using the WHO classification of the clinical stages of KS skin lesions, the nodular stage was most frequently detected in patients with classic or iatrogenic KS, whereas the number of patchy/plaque and nodular stages was similar in patients with AIDS-related KS in our cohort (Table 2). The histological findings of KS were similar among clinical types and viral genotypes (Figure 1).

**TABLE 2:** Skin lesions and stage classification.

Clinical type		Classic	AIDS-related	Iatrogenic
Skin lesions in total cases		18/18	7/10	11/12
Stage	Patchy-Plaque	4/18 (22.2%)	4/7 (57.1%)	4/11 (36.3%)
	Nodular	14/18 (77.8%)	3/7 (42.9%)	7/11 (63.6%)

### KSHV genotype of KS in Okinawa

**FIGURE 2:**
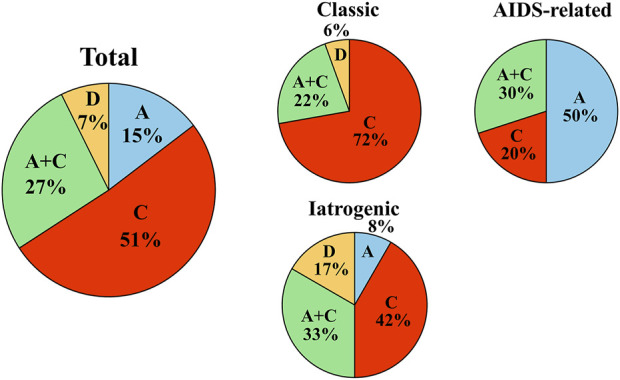
KSHV genotype prevalence in Okinawa, Japan. KSHV genotype prevalence in Okinawa, Japan. Pie charts indicate the KSHV genotype prevalence of all patients with KS in the cohort (left), genotype prevalence among patients with classic KS (upper middle), genotype prevalence among patients with AIDS-related KS (right), and genotype prevalence among patients with iatrogenic KS (lower middle) in Okinawa, Japan. Among all patients with KS in the cohort, the most prevalent viral genotype is single genotype C, followed by dual genotypes A and C. The most prevalent KSHV genotype in classic KS is genotype C, although single genotype A is most frequent in patients with AIDS-related KS.

KSHV genotyping using K1 revealed that 78% of all patients with KS in Okinawa were infected with genotype C, of which 51% were infected with single genotype C and 27% had dual infection of genotypes A and C (Figure 2). In contrast, the prevalence of single infection with either genotype A or D was low in the total patients with KS in Okinawa (Figure 2). To the best of our knowledge, this study is the first to identify genotype D in Japanese patients with KS. Genotype B was not detected in our cohort.

### KSHV K1 genotype and clinical presentation

Next, we investigated the association between the KSHV genotype and clinical presentation. Single genotype C or dual genotypes A and C infections were detected in 94% of patients with classic KS, with 72% of them infected with single genotype C (Figure 2). However, patients with AIDS-related KS tended to have genotype A infection (80%), which was either single genotype A (50%) or dual genotypes A and C (30%) (Figure 2). Among AIDS-related KS patients, single genotype C accounted for only 20% of infections. Similarly to classic KS, most patients with iatrogenic KS were infected with genotype C (75%), with 42% infected with single genotype C. The proportion of genotype D (17%) was highest among the patients with iatrogenic KS. In our cohort, dual infection with genotypes A and C accounted for 20%–30% of any clinical types.

### KSHV K15 genotype and clinical presentation

According to K15 genotyping, genotype M was more prevalent than genotype P in patients with KS in Okinawa. Regarding the association between the K15 subtype and KS clinical type, genotype M was frequent in classic and iatrogenic KSs, whereas genotype P was predominant in AIDS-related KS (Table 1).

### Association between KSHV K1 and K15 genotypes

Next, we evaluated the association between the K1 and K15 genotypes and found that a single genotype A was associated with genotype P, whereas a single genotype C was associated with genotype M; however, a statistical correlation could not be established owing to the limited cohort size (Table 3). Patients with dual genotypes A and C had similar frequencies of genotypes M and genotype P.

**TABLE 3:** The association between the K1 and K15 genotypes.

K1 genotype		A	A + C	C	D
Number of cases^a^		5 (6)	7 (12)	14 (20)	1 (3)
K15	P	4	3	4	0
	M	1	4	10	1

^a^ The number of cases indicates that the cases could be successfully detected by K15 PCR. The numbers in parentheses indicate the cases examined.

### Association between KSHV genotypes and skin and visceral lesions

In our cohort, all patients with classic KS exhibited lesions limited to the skin without any visceral lesions (Table 1). Examining skin lesions of patients with classic KS, genotype C was the most prevalent, with genotype A comprising a small proportion (Table 4). However, skin lesions of patients with AIDS-related or iatrogenic KS revealed a similar prevalences of genotypes A and C (Table 4). In terms of visceral lesions, two patients with AIDS-related KS developed lung or lymph node lesions, both of whom were infected with a single genotype A. Colonic lesions were detected in one patient with AIDS-related KS, identified as a dual infection of genotypes A and C. One patient with iatrogenic KS developed a lymph node lesion infected with the genotype C.

**TABLE 4:** Skin lesions and K1 genotyping.

K1 genotype	Single A	Dual A + C	Single C	Single D
Classic	0	4	13	1
AIDS-related	3	2	2	0
Iatrogenic	1	5	3	2

## Discussion

In this study, we found that classic KS was the dominant clinical type of KS in patients from Okinawa. Both classic and iatrogenic KS were detected in older patients, whereas AIDS-related KS was identified in younger patients. Male patients were predominantly diagnosed with any clinical types of KS. Half of the patients with classic or iatrogenic KS were from the Miyako islands where classic KS is known to be endemic. In Okinawa, genotype C was the predominant genotype in all patients with KS. Notably, half of the patients with KS were infected with single genotype C, while 27% were infected with dual genotypes A and C. Conversely, single genotype A was detected in a small proportion of our cohort, whereas genotype D was extremely rare. To the best of our knowledge, our study is the first to identify genotype D in Japanese patients with KS.

Regarding the association between viral genotype and clinical type, over 90% of patients with classic KS in Okinawa were infected with genotype C, with 72% infected with single genotype C. Conversely, 80% of the patients with AIDS-related KS were associated with genotype A, of which half of the patients were infected with single genotype A. Iatrogenic KS exhibited a genotype prevalence similar to that of classic KS.

Given that KSHV K1 and K15 exhibit high polymorphism, the sequence variability of K1 is utilized for genotyping [7, 14, 23]. Although genotype C is the predominant genotype in Japan, genotype A is frequently detected in patients with AIDS [24]. Okinawa, southwestern islands of Japan, is recognized as an endemic area for classic KS [20, 22]. In the current study, we confirmed that genotype C was the predominant genotype in patients with KS in Okinawa, which is consistent with the results of KSHV genome analysis in Miyako, Okinawa [22]. Our findings suggest that genotype A infection is more prevalent than genotype C infection in patients with AIDS-related KS in Okinawa. A similar KSHV genotype prevalence has been observed in mainland Japan [24].

Regarding the K15 genotyping among patients with KS in Okinawa, genotype M was more prevalent than genotype P. Although genotype M accounted for most cases of classic and iatrogenic KS in Okinawa, genotype P constituted the majority of AIDS-related KS cases. In terms of the geographic distribution of K15 subtypes, genotype P is known to be prevalent in large parts of Europe, including France, Greece, Russia, and Ireland [15, 25, 26]. Although Lacoste et al. reported that genotype M has higher allelic rates in Central and Western Africa, others have argued that genotype P is prevalent in Zambia and Cameroon [25, 27, 28]. Genotype M has been reported in relatively high proportions in the USA and Eastern Asia, including South Korea and Taiwan [9]. In our cohort, the proportion of patients infected with genotype M was greater than that infected with genotype P. Given that Okinawa islands are located in the East China Sea, especially Miyako island, where classic KS is endemic, and is located between Taiwan and mainland Japan, the high proportion of genotype M in Okinawa may be associated with both the geographical location and population migration in ancient times.

Regarding the association between viral genotypes K1 and K15, genotype A of K1 was related to genotype P of K15, whereas genotype C of K1 was related to genotype M of K15 in our cohort. These results are inconsistent with those reported by Lacoste et al. who showed that the majority of genotype C was linked to genotype P and genotype A was predominantly associated with genotype M. In our cohort, genotype A/P and C/M tended to be associated with AIDS-related and classic KS, respectively. However, Lacoste et al. reported that genotypes C/P and A/M were associated with AIDS-related, multiple Castleman disease and primary effusion lymphoma. Given that the clinical presentations of our cohort markedly differed from that of Lacoste et al., direct comparison could not be performed. Nevertheless, these results suggest that the genomic structures of KSHV from Okinawa are different from those detected in France and certain African countries.

Regarding the association between viral genotype and skin lesions, most skin lesions detected in patients with classic KS in our cohort were linked to genotype C. However, skin lesions in patients with AIDS-related and iatrogenic KS exhibited identical frequencies of genotypes A and C. Classic KS usually presents as cutaneous lesions on the extremities and rarely involves visceral organs. However, AIDS-related KS arises not only in cutaneous tissues but also in mucous tissues and visceral organs, with the possibility of developing multiple visceral lesions [1–3]. In the current study, all patients with classic KS exhibited lesions limited to the skin and had either a single genotype C or dual genotypes A and C; there were no patients with classic KS of single genotype A in our cohort. We observed that three out of ten patients with AIDS-related KS and one out of twelve patients with iatrogenic KS developed visceral lesions. These three patients with AIDS-related KS presented with visceral lesions, all of whom were infected with genotype A, and, developed lymph node, intestinal, and pulmonary lesions. One patient with iatrogenic KS, infected with genotype C, was treated with prednisolone for nephrotic syndrome and developed lymph node involvement. Although a single genotype C was primarily detected in the cutaneous lesions in our study, the presence of a patient with iatrogenic KS of genotype C suggested that visceral lesions may develop when host immunity is impaired. Although we observed that patients with classic and iatrogenic KS infected with genotype C demonstrated greater progression to the advanced stage of cutaneous lesions than those with AIDS-related KS, these patients did not experience visceral organ involvement, and manifestations were limited to the skin, despite some patients with AIDS-related KS infected with genotype A developing visceral lesions.

Host immunity against KSHV is a key factor in KS development [1–3]. Immunocompromised patients with AIDS develop AIDS-related KS as an opportunistic infection. In the present study, all patients with AIDS had a CD4 cell count of <200/μL, except for one patient, with half of the patients (five cases) exhibiting a count of <50/μL, which is associated with a substantially increased risk of opportunistic infection (Supplementary Table S1). Classic KS frequently occurs in older individuals, implying that age-related decline in host immunity is related to the development of classic KS. KSHV infection induces both innate and adaptive immunity [29, 30]. Sirianni et al. reported the preferential infiltration of CD8 cells in patients with both AIDS-related and classic KS, and also showed increased interferon (IFN)-γ production in peripheral blood mononuclear cells. The authors concluded that cell-mediated immunity was induced by Th1 cells (IFN-γ) against KS [31]. In this study, the reduced number of CD4 cells in patients with AIDS may have led to the attenuation of cell-mediated immunity by Th1 cells. Given that genotype C was predominantly detected in patients with classic KS and genotype A was mainly detected in those with AIDS-related KS in this study, differences in the number of immune cells, especially CD4 cells, between genotypes A and C may be attributed to the potency of the host immune response to KS. It should be noted that aging is a known factor contributing to reduced immunity, and other factors such as poor nutrition, renal and liver dysfunctions, and diabetes mellitus can also impact the immune status. In our cohort, nearly half of the patients with classic KS (n = 9) had hypoalbuminemia, and some had reduced renal function and diabetes mellitus (Supplementary Table S1). The findings of the current study suggest that the KSHV genotype in Okinawa differs from that detected in other countries, and is related to the KSHV geographic distribution and population migration. Our data also suggest that the viral genotype in Okinawa is associated with clinical presentations. To elucidate the distinctiveness of the KSHV genotype in Okinawa and the accurate correlation between the KSHV genotype and clinical presentation, a study including a larger cohort accounting for the immune status of patients with KS is needed.

## Data Availability

The original contributions presented in the study are included in the article/Supplementary Material, further inquiries can be directed to the corresponding author.

## Abbreviations

KS, Kaposi’s sarcoma; KSHV, Kaposi’s sarcoma associated herpesvirus; AIDS, Acquired immunodeficiency syndrome; ORFs, Open reading frames; FFPE, Formalin fixed paraffin embedded; VR, Variable region; HIV, Human immunodeficiency virus.
