# Supplementary material for: Association between Kaposi’s sarcoma-associated herpesvirus genotype and clinical types
Source: Pathol Oncol Res. 2025 Apr 28;31:1612009. doi: 10.3389/pore.2025.1612009 (PMC12066305; doi:10.3389/pore.2025.1612009)
Supplement: Supplementary file 1 [file Table1.docx]

**Supplemental Table 1. Baseline characteristics of patients with KS**

| No | Age | Sex | Clinical type | K1 | K15 | WBC  (10^3^/µL) | Lymphocyte　(10^9^/µL) | CD4  (cells/µL) | | Alb  (g/dL) | BUN  (mg/dL) | Cre  (mg/dL) | GOT  (IU/L) | GPT  (IU/L) | ALP  (IU/L) |
| --- | --- | --- | --- | --- | --- | --- | --- | --- | --- | --- | --- | --- | --- | --- | --- |
| 2 | 80 | M | Classic | A+C | - | 6.7 | 1.47 | | - | 3.3 | 11 | 1.2 | 78 | 54 | 125 |
| 5 | 79 | M | Classic | C | M | 7.0 | 1.72 | | - | 4 | 16 | 0.8 | 18 | 17 | 292 |
| 6 | 85 | M | Classic | C | - | 5.4 | 0.87 | | - | 3.5 | 14 | 0.7 | 23 | 12 | - |
| 9 | 88 | M | Classic | C | - | 5.7 | 1.39 | | - | 3.6 | 14 | 0.6 | 16 | 12 | 233 |
| 11 | 78 | M | Classic | C | - | 6.7 | 1.97 | | - | 4.1 | 22 | 1.2 | 29 | 35 | 125 |
| 12 | 91 | F | Classic | D | - | 9.3 | 1.64 | | - | 3.1 | 15 | 0.8 | 11 | 7 | 349 |
| 14 | 81 | F | Classic | C | P | - | - | | - | - | - | - | - | - | - |
| 15 | 58 | M | Classic | C | P | 6.8 | - | | - | 3.9 | 18 | 0.9 | 27 | 20 | 242 |
| 16 | 65 | M | Classic | C | M | 7.6 | 0.68 | | - | 4.9 | 15 | 0.6 | 26 | 25 | 262 |
| 19 | 92 | M | Classic | A+C | - | 8.9 | 1.82 | | - | - | - | - | 39 | 27 | 235 |
| 20 | 93 | F | Classic | A+C | M | - | - | | - | - | - | - | - | - | - |
| 21 | 86 | M | Classic | C | - | 6.7 | 1.61 | | - | 4.1 | 44 | 1.7 | 14 | 12 | 212 |
| 23 | 80 | M | Classic | C | M | 5.3 | 1.25 | | - | 4.2 | 12 | 1.1 | 15 | 15 | 153 |
| 26 | 70 | M | Classic | C | P | 6.3 | 2.14 | | - | 3.9 | 20 | 1.2 | 20 | 7 | 240 |
| 28 | 64 | M | Classic | A+C | M | 6.2 | 1.84 | | - | - | - | - | - | - | - |
| 36 | 93 | F | Classic | C | M | 6.7 | 1.63 | | - | 4 | 11 | 0.6 | 33 | 14 | 432 |
| 37 | 72 | M | Classic | C | M | 5.3 | 2.21 | | - | 3.8 | 24 | 1.5 | 26 | 20 | 52 |
| 40 | 84 | M | Classic | C | M | - | - | | - | - | - | - | - | - | - |
| 41 | 88 | M | Classic | C | M | 16.3 | 0.93 | | - | - | 32 | 1.6 | 13 | 12 | 46 |
| 7 | 42 | M | AIDS-related | A+C | P | 4.8 | 2.43 | | 380 | 3.6 | 9 | 0.4 | 49 | 93 | 181 |
| 8 | 55 | M | AIDS-related | C | P | 3.2 | 0.62 | | 115 | 2.9 | 5 | 0.5 | 25 | 31 | 279 |
| 17 | 40 | M | AIDS-related | A+C | M | 4.4 | 1.54 | | 174 | 3.5 | 13 | 0.7 | 37 | 85 | 293 |
| 22 | 40 | M | AIDS-related | A | M | 4.4 | 1.36 | | 157 | 3 | 4 | 0.5 | 63 | 57 | - |
| 25 | 35 | M | AIDS-related | A | P | 2.7 | 0.37 | | 34 | 3 | 8 | 0.7 | 16 | 8 | 214 |
| 27 | 36 | M | AIDS-related | A+C | P | 3.6 | 0.80 | | 111 | 4.4 | 14 | 0.7 | 33 | 32 | 1029 |
| 31 | 54 | M | AIDS-related | A | P | 2.3 | 0.53 | | 9 | - | 10 | 0.7 | 20 | 18 | 407 |
| 32 | 31 | M | AIDS-related | C | - | 4.2 | 0.73 | | 13 | 3.9 | 9 | 0.8 | 26 | 23 | 350 |
| 38 | 25 | M | AIDS-related | A | P | 4.1 | 1.49 | | 26 | 4.7 | 8 | 0.9 | 14 | 15 | 56 |
| 39 | 34 | M | AIDS-related | A | P | 6.2 | 2.28 | | 23 | 4.6 | 17 | 1.0 | 34 | 64 | 97 |
| 1 | 72 | F | Iatrogenic | A+C | P | 14.9 | 1.34 | | - | 3.7 | 27 | 1.0 | 33 | 36 | 150 |
| 3 | 80 | M | Iatrogenic | C | - | 4.5 | 1.10 | | - | 3.9 | 12 | 0.6 | 16 | 14 | 159 |
| 4 | 73 | M | Iatrogenic | A | - | 8.5 | 2.60 | | - | 4.5 | 30 | 1.5 | 16 | 10 | 408 |
| 10 | 62 | M | Iatrogenic | C | - | 17.2 | 0.34 | | - | 4 | 23 | 0.9 | 20 | 41 | 244 |
| 13 | 70 | M | Iatrogenic | D | - | 6.0 | 0.47 | | - | 2.9 | 15 | 1.0 | 13 | 56 | 293 |
| 18 | 89 | F | Iatrogenic | A+C | - | - | - | | - | - | - | - | - | - | - |
| 24 | 69 | M | Iatrogenic | A+C | M | 7.9 | 0.96 | | - | 4.1 | 31 | 1.3 | 23 | 9 | 211 |
| 29 | 70 | M | Iatrogenic | A+C | - | 8.5 | 1.36 | | - | 4.1 | 12 | 1.0 | 17 | 14 | 249 |
| 30 | 73 | M | Iatrogenic | C | M | - | - | | - | - | - | - | - | - | - |
| 33 | 81 | M | Iatrogenic | D | M | 29.3 | 3.96 | | - | 3.9 | 27 | 1.6 | 19 | 8 | 150 |
| 34 | 83 | M | Iatrogenic | C | M | 9 | 0.84 | | - | 4 | 27 | 1.1 | 18 | 24 | 129 |
| 35 | 74 | F | Iatrogenic | C | M | - | - | | - | - | - | - | - | - | - |
